# {5-Chloro-2-[(4-chloro­benzyl­idene)­amino]­phen­yl}(phen­yl)methanone

**DOI:** 10.1107/S1600536812004655

**Published:** 2012-02-10

**Authors:** Muhammad Aslam, Itrat Anis, Nighat Afza, Shazia Yasmeen, Sammer Yousuf

**Affiliations:** a Pharmaceutical Research Centre, PCSIR Laboratories Complex, Karachi, Pakistan; b Department of Chemistry, University of Karachi, Karachi, Pakistan; c HEJ Research Institute of Chemistry, International Center for Chemical and Biological Sciences, University of Karachi, Karachi 75270, Pakistan

## Abstract

In the title compound, C_20_H_13_Cl_2_NO, the C=N bond adopts an *E* conformation. The chloro-substituted rings form a dihedral angle of 11.99 (9)° with each other and form dihedral angles of 74.95 (9) and 83.26 (10)° with the unsubstituted ring. In the crystal, mol­ecules are connected into dimers by pairs of weak C—H⋯O hydrogen bonds and the dimers are arranged in columns parallel to the *a* axis.

## Related literature

For the biological activities of Schiff base compounds, see: Solomon & Lowery (1993 ▶). For related structures, see: Aslam *et al.* (2011*a* ▶,*b* ▶); Zeb & Yousuf (2011 ▶).
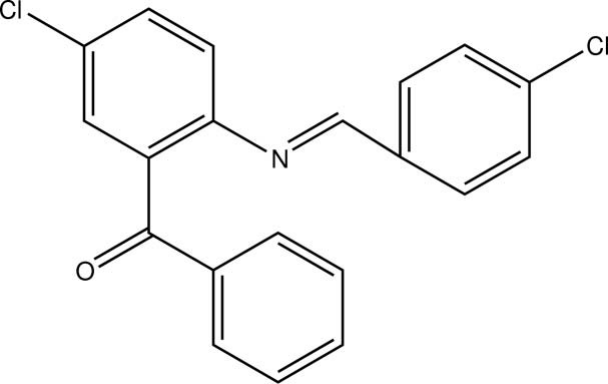


## Experimental

### 

#### Crystal data

C_20_H_13_Cl_2_NO
*M* *_r_* = 354.21
Triclinic, 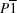

*a* = 7.2283 (4) Å
*b* = 10.2301 (5) Å
*c* = 11.9079 (6) Å
α = 100.929 (1)°
β = 97.318 (1)°
γ = 91.360 (1)°
*V* = 856.49 (8) Å^3^
*Z* = 2
Mo *K*α radiation
μ = 0.38 mm^−1^
*T* = 273 K
0.39 × 0.14 × 0.10 mm

#### Data collection

Bruker SMART APEX CCD diffractometer
Absorption correction: multi-scan (*SADABS*; Bruker, 2000 ▶) *T*_min_ = 0.865, *T*_max_ = 0.963
9733 measured reflections
3186 independent reflections
2388 reflections with *I* > 2σ(*I*)
*R*_int_ = 0.025

#### Refinement

*R*[*F* ^2^ > 2σ(*F* ^2^)] = 0.037
*wR*(*F* ^2^) = 0.102
*S* = 1.02
3186 reflections
217 parameters
H-atom parameters constrained
Δρ_max_ = 0.18 e Å^−3^
Δρ_min_ = −0.23 e Å^−3^

### 

Data collection: *SMART* (Bruker, 2000 ▶); cell refinement: *SAINT* (Bruker, 2000 ▶); data reduction: *SAINT*; program(s) used to solve structure: *SHELXS97* (Sheldrick, 2008 ▶); program(s) used to refine structure: *SHELXL97* (Sheldrick, 2008 ▶); molecular graphics: *SHELXTL* (Sheldrick, 2008 ▶); software used to prepare material for publication: *SHELXTL*, *PARST* (Nardelli, 1995 ▶) and *PLATON* (Spek, 2009 ▶).

## Supplementary Material

Crystal structure: contains datablock(s) global, I. DOI: 10.1107/S1600536812004655/lh5403sup1.cif

Structure factors: contains datablock(s) I. DOI: 10.1107/S1600536812004655/lh5403Isup2.hkl

Supplementary material file. DOI: 10.1107/S1600536812004655/lh5403Isup3.cml

Additional supplementary materials:  crystallographic information; 3D view; checkCIF report

## Figures and Tables

**Table 1:** Hydrogen-bond geometry (Å, °)

*D*—H⋯*A*	*D*—H	H⋯*A*	*D*⋯*A*	*D*—H⋯*A*
C9—H9*A*⋯O1^i^	0.93	2.52	3.426 (2)	164

Symmetry code: (i) 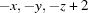.

**Fig. 1.**
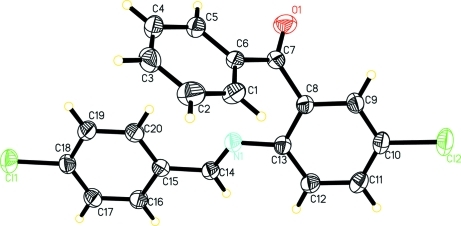
The molecular structure of (I) with displacement ellipsoids drawn at 30% probability level.

**Fig. 2.**
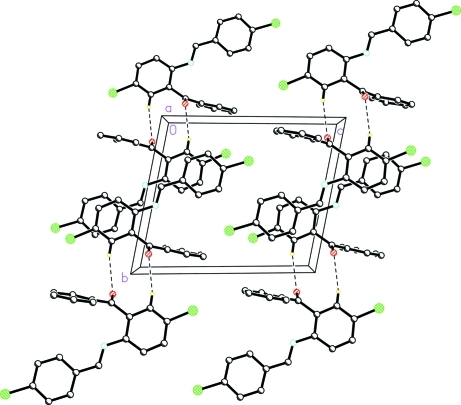
The crystal packing of (I) with hydrogen bonds shown as dashed lines. Only hydrogen atoms involved in hydrogen bonding are shown.

## supplementary crystallographic information

### Comment

Schiff bases are well known ligands in synthetic chemistry and have a wide range of bilogical activities (Solomon & Lowery, 1993). The title compound (I) (Fig. 1) is a schiff base structurally similar to our recently published compound {5-chloro-2-[(4-nitrobenzylidene)amino]phenyl}(phenyl)methanone (Aslam *et al.*, 2011*b*) with a difference that the nitro group on the benzene ring (C15-C20) is replaced by a chloro group in the title compound. The torsion angle of the E configurated double bond (C═N, 1.253 (2) Å) is 177.31 (15)° for C13-N1-C14-C15. The bond lengths and angles are similar to those in the previously reported structural analogue (Aslam *et al.*, 2011*b*). We have published other crystal structures of this type of compound (Aslam *et al.*, 2011*a*; Zeb & Yousuf, 2011). In the crystal, molecules are linked into dimers *via* weak C9—H9A···O1^i^ intermolecular hydrogen bonds (symmetry code as in Table 1) and arranged in columns parallel to the *a* axis.

### Experimental

The synthesis of title compound was carried out by refluxing a mixture of 4-chlorobenzaldehyde (1 mol) and 2-amino-5-chlorobenzophenone (1 mol) in ethanol (50 ml) along with 3 drops of conc. H_2_SO_4_ for 5 h at 343 K. After cooling, the mixture was concentrated to one third under reduced pressure. The concentrated reaction mixture was kept at room temperature and yellow crystals were obtained after nine days. The crystalline product was collected, washed with methanol and dried to afford the title compound in 85% yield. Slow evaporation of a methanol solution afforded yellow crystals which were suitable for single-crystal X-ray diffraction studies. All chemicals were purchased from Sigma-Aldrich.

### Refinement

H atoms were positioned geometrically with C—H = 0.93 Å, and constrained to ride on their parent atoms with *U*_iso_(H)= 1.2*U*_eq_(CH).

### Crystal data

**Table:**

C_20_H_13_Cl_2_NO	*Z* = 2
*M**_r_* = 354.21	*F*(000) = 364
Triclinic, *P*1	*D*_x_ = 1.373 Mg m^−^^3^
Hall symbol: -P 1	Mo *K*α radiation, λ = 0.71073 Å
*a* = 7.2283 (4) Å	Cell parameters from 2693 reflections
*b* = 10.2301 (5) Å	θ = 2.4–25.0°
*c* = 11.9079 (6) Å	µ = 0.38 mm^−^^1^
α = 100.929 (1)°	*T* = 273 K
β = 97.318 (1)°	Block, yellow
γ = 91.360 (1)°	0.39 × 0.14 × 0.10 mm
*V* = 856.49 (8) Å^3^	

### Data collection

**Table:**

Bruker SMART APEX CCD diffractometer	3186 independent reflections
Radiation source: fine-focus sealed tube	2388 reflections with *I* > 2σ(*I*)
Graphite monochromator	*R*_int_ = 0.025
ω scans	θ_max_ = 25.5°, θ_min_ = 1.8°
Absorption correction: multi-scan (*SADABS*; Bruker, 2000)	*h* = −8→8
*T*_min_ = 0.865, *T*_max_ = 0.963	*k* = −12→12
9733 measured reflections	*l* = −14→14

### Refinement

**Table:**

Refinement on *F*^2^	Primary atom site location: structure-invariant direct methods
Least-squares matrix: full	Secondary atom site location: difference Fourier map
*R*[*F*^2^ > 2σ(*F*^2^)] = 0.037	Hydrogen site location: inferred from neighbouring sites
*wR*(*F*^2^) = 0.102	H-atom parameters constrained
*S* = 1.02	*w* = 1/[σ^2^(*F*_o_^2^) + (0.0503*P*)^2^ + 0.1325*P*] where *P* = (*F*_o_^2^ + 2*F*_c_^2^)/3
3186 reflections	(Δ/σ)_max_ = 0.001
217 parameters	Δρ_max_ = 0.18 e Å^−^^3^
0 restraints	Δρ_min_ = −0.23 e Å^−^^3^

### Special details

**Table:**

Geometry. All e.s.d.'s (except the e.s.d. in the dihedral angle between two l.s. planes) are estimated using the full covariance matrix. The cell e.s.d.'s are taken into account individually in the estimation of e.s.d.'s in distances, angles and torsion angles; correlations between e.s.d.'s in cell parameters are only used when they are defined by crystal symmetry. An approximate (isotropic) treatment of cell e.s.d.'s is used for estimating e.s.d.'s involving l.s. planes.
Refinement. Refinement of *F*^2^ against ALL reflections. The weighted *R*-factor *wR* and goodness of fit *S* are based on *F*^2^, conventional *R*-factors *R* are based on *F*, with *F* set to zero for negative *F*^2^. The threshold expression of *F*^2^ > σ(*F*^2^) is used only for calculating *R*-factors(gt) *etc*. and is not relevant to the choice of reflections for refinement. *R*-factors based on *F*^2^ are statistically about twice as large as those based on *F*, and *R*- factors based on ALL data will be even larger.

### Fractional atomic coordinates and isotropic or equivalent isotropic displacement parameters (Å^2^)

**Table:**

	*x*	*y*	*z*	*U*_iso_*/*U*_eq_	
Cl1	0.21952 (9)	0.72197 (6)	0.48534 (5)	0.0772 (2)	
Cl2	0.26120 (8)	0.21797 (7)	1.37525 (4)	0.0772 (2)	
O1	−0.06787 (19)	0.12992 (15)	0.92259 (12)	0.0703 (4)	
N1	0.2217 (2)	0.43376 (15)	0.94686 (13)	0.0534 (4)	
C1	0.3968 (3)	0.1501 (2)	0.85494 (17)	0.0593 (5)	
H1B	0.4588	0.1680	0.9300	0.071*	
C2	0.4979 (3)	0.1263 (2)	0.7624 (2)	0.0747 (7)	
H2A	0.6275	0.1257	0.7753	0.090*	
C3	0.4060 (4)	0.1037 (2)	0.65140 (19)	0.0715 (6)	
H3A	0.4740	0.0911	0.5893	0.086*	
C4	0.2164 (3)	0.0997 (2)	0.63220 (17)	0.0640 (6)	
H4A	0.1549	0.0833	0.5570	0.077*	
C5	0.1152 (3)	0.12004 (19)	0.72373 (16)	0.0542 (5)	
H5A	−0.0146	0.1154	0.7100	0.065*	
C6	0.2049 (2)	0.14729 (16)	0.83616 (14)	0.0448 (4)	
C7	0.0923 (3)	0.17452 (17)	0.93309 (15)	0.0465 (4)	
C8	0.1764 (2)	0.25790 (17)	1.04654 (14)	0.0443 (4)	
C9	0.1823 (2)	0.20517 (19)	1.14596 (15)	0.0507 (4)	
H9A	0.1410	0.1174	1.1419	0.061*	
C10	0.2499 (3)	0.2842 (2)	1.25056 (15)	0.0520 (5)	
C11	0.3079 (3)	0.4151 (2)	1.25924 (16)	0.0566 (5)	
H11A	0.3517	0.4674	1.3309	0.068*	
C12	0.3006 (3)	0.4679 (2)	1.16108 (16)	0.0560 (5)	
H12A	0.3397	0.5564	1.1668	0.067*	
C13	0.2355 (2)	0.39071 (17)	1.05307 (14)	0.0453 (4)	
C14	0.2384 (2)	0.55370 (18)	0.93892 (15)	0.0483 (4)	
H14A	0.2559	0.6186	1.0061	0.058*	
C15	0.2314 (2)	0.59480 (17)	0.82787 (15)	0.0448 (4)	
C16	0.2588 (3)	0.72787 (18)	0.82228 (16)	0.0522 (5)	
H16A	0.2787	0.7915	0.8902	0.063*	
C17	0.2569 (3)	0.76737 (19)	0.71788 (17)	0.0550 (5)	
H17A	0.2763	0.8568	0.7151	0.066*	
C18	0.2260 (3)	0.6728 (2)	0.61789 (16)	0.0519 (5)	
C19	0.1977 (3)	0.5396 (2)	0.62006 (17)	0.0595 (5)	
H19A	0.1768	0.4766	0.5518	0.071*	
C20	0.2007 (3)	0.50153 (19)	0.72493 (16)	0.0559 (5)	
H20A	0.1819	0.4119	0.7272	0.067*	

### Atomic displacement parameters (Å^2^)

**Table:**

	*U*^11^	*U*^22^	*U*^33^	*U*^12^	*U*^13^	*U*^23^
Cl1	0.1037 (5)	0.0795 (4)	0.0541 (3)	−0.0031 (3)	0.0087 (3)	0.0294 (3)
Cl2	0.0884 (4)	0.1037 (5)	0.0477 (3)	0.0080 (3)	0.0099 (3)	0.0346 (3)
O1	0.0583 (9)	0.0847 (10)	0.0620 (9)	−0.0190 (8)	0.0089 (7)	0.0014 (7)
N1	0.0718 (11)	0.0451 (9)	0.0437 (9)	−0.0017 (7)	0.0051 (7)	0.0119 (7)
C1	0.0574 (12)	0.0635 (13)	0.0513 (11)	−0.0004 (10)	0.0015 (9)	0.0013 (9)
C2	0.0594 (13)	0.0807 (16)	0.0788 (16)	−0.0021 (11)	0.0199 (12)	−0.0038 (12)
C3	0.0960 (18)	0.0620 (13)	0.0584 (13)	−0.0072 (12)	0.0325 (12)	0.0032 (10)
C4	0.0888 (16)	0.0603 (13)	0.0429 (11)	−0.0038 (11)	0.0063 (10)	0.0130 (9)
C5	0.0624 (12)	0.0537 (11)	0.0463 (10)	0.0001 (9)	0.0008 (9)	0.0136 (8)
C6	0.0529 (10)	0.0389 (9)	0.0415 (9)	−0.0020 (8)	0.0026 (8)	0.0081 (7)
C7	0.0496 (10)	0.0441 (10)	0.0461 (10)	−0.0027 (8)	0.0024 (8)	0.0125 (8)
C8	0.0453 (9)	0.0475 (10)	0.0407 (9)	0.0010 (8)	0.0077 (7)	0.0091 (8)
C9	0.0518 (10)	0.0567 (11)	0.0474 (10)	−0.0009 (9)	0.0094 (8)	0.0181 (9)
C10	0.0489 (10)	0.0710 (13)	0.0404 (10)	0.0094 (9)	0.0077 (8)	0.0196 (9)
C11	0.0618 (12)	0.0670 (13)	0.0385 (10)	0.0059 (10)	0.0053 (8)	0.0044 (9)
C12	0.0702 (13)	0.0476 (11)	0.0475 (11)	−0.0010 (9)	0.0056 (9)	0.0044 (9)
C13	0.0490 (10)	0.0479 (10)	0.0398 (9)	0.0030 (8)	0.0074 (8)	0.0092 (8)
C14	0.0542 (11)	0.0454 (11)	0.0445 (10)	0.0002 (8)	0.0061 (8)	0.0072 (8)
C15	0.0428 (9)	0.0440 (10)	0.0482 (10)	0.0010 (7)	0.0063 (8)	0.0107 (8)
C16	0.0638 (12)	0.0421 (10)	0.0499 (11)	0.0017 (8)	0.0069 (9)	0.0076 (8)
C17	0.0655 (12)	0.0439 (10)	0.0581 (12)	0.0002 (9)	0.0082 (9)	0.0163 (9)
C18	0.0523 (10)	0.0586 (12)	0.0480 (11)	0.0010 (9)	0.0063 (8)	0.0190 (9)
C19	0.0775 (13)	0.0526 (12)	0.0459 (11)	−0.0047 (10)	0.0041 (9)	0.0069 (9)
C20	0.0710 (13)	0.0426 (10)	0.0539 (11)	−0.0041 (9)	0.0052 (9)	0.0119 (9)

### Geometric parameters (Å, º)

**Table:**

Cl1—C18	1.7406 (18)	C9—C10	1.374 (3)
Cl2—C10	1.7389 (18)	C9—H9A	0.9300
O1—C7	1.217 (2)	C10—C11	1.375 (3)
N1—C14	1.253 (2)	C11—C12	1.373 (3)
N1—C13	1.409 (2)	C11—H11A	0.9300
C1—C6	1.375 (3)	C12—C13	1.394 (2)
C1—C2	1.385 (3)	C12—H12A	0.9300
C1—H1B	0.9300	C14—C15	1.457 (2)
C2—C3	1.377 (3)	C14—H14A	0.9300
C2—H2A	0.9300	C15—C16	1.386 (2)
C3—C4	1.359 (3)	C15—C20	1.393 (3)
C3—H3A	0.9300	C16—C17	1.377 (3)
C4—C5	1.375 (3)	C16—H16A	0.9300
C4—H4A	0.9300	C17—C18	1.374 (3)
C5—C6	1.385 (2)	C17—H17A	0.9300
C5—H5A	0.9300	C18—C19	1.379 (3)
C6—C7	1.483 (2)	C19—C20	1.375 (3)
C7—C8	1.501 (2)	C19—H19A	0.9300
C8—C9	1.387 (2)	C20—H20A	0.9300
C8—C13	1.399 (2)		
			
C14—N1—C13	123.34 (16)	C12—C11—C10	119.37 (17)
C6—C1—C2	120.21 (19)	C12—C11—H11A	120.3
C6—C1—H1B	119.9	C10—C11—H11A	120.3
C2—C1—H1B	119.9	C11—C12—C13	120.96 (18)
C3—C2—C1	119.8 (2)	C11—C12—H12A	119.5
C3—C2—H2A	120.1	C13—C12—H12A	119.5
C1—C2—H2A	120.1	C12—C13—C8	118.58 (16)
C4—C3—C2	120.3 (2)	C12—C13—N1	126.03 (17)
C4—C3—H3A	119.9	C8—C13—N1	115.39 (15)
C2—C3—H3A	119.9	N1—C14—C15	122.11 (17)
C3—C4—C5	120.1 (2)	N1—C14—H14A	118.9
C3—C4—H4A	119.9	C15—C14—H14A	118.9
C5—C4—H4A	119.9	C16—C15—C20	118.33 (17)
C4—C5—C6	120.57 (19)	C16—C15—C14	120.65 (16)
C4—C5—H5A	119.7	C20—C15—C14	121.01 (16)
C6—C5—H5A	119.7	C17—C16—C15	121.11 (17)
C1—C6—C5	118.97 (17)	C17—C16—H16A	119.4
C1—C6—C7	121.64 (16)	C15—C16—H16A	119.4
C5—C6—C7	119.38 (16)	C18—C17—C16	119.08 (17)
O1—C7—C6	121.13 (16)	C18—C17—H17A	120.5
O1—C7—C8	119.03 (16)	C16—C17—H17A	120.5
C6—C7—C8	119.84 (15)	C17—C18—C19	121.44 (17)
C9—C8—C13	120.33 (16)	C17—C18—Cl1	119.46 (15)
C9—C8—C7	119.33 (16)	C19—C18—Cl1	119.10 (15)
C13—C8—C7	120.17 (15)	C20—C19—C18	118.84 (18)
C10—C9—C8	119.20 (17)	C20—C19—H19A	120.6
C10—C9—H9A	120.4	C18—C19—H19A	120.6
C8—C9—H9A	120.4	C19—C20—C15	121.20 (17)
C9—C10—C11	121.53 (17)	C19—C20—H20A	119.4
C9—C10—Cl2	119.45 (16)	C15—C20—H20A	119.4
C11—C10—Cl2	119.01 (15)		
			
C6—C1—C2—C3	1.9 (3)	C10—C11—C12—C13	0.0 (3)
C1—C2—C3—C4	−2.4 (4)	C11—C12—C13—C8	0.3 (3)
C2—C3—C4—C5	0.8 (3)	C11—C12—C13—N1	180.00 (17)
C3—C4—C5—C6	1.3 (3)	C9—C8—C13—C12	0.3 (3)
C2—C1—C6—C5	0.2 (3)	C7—C8—C13—C12	175.50 (16)
C2—C1—C6—C7	−178.95 (19)	C9—C8—C13—N1	−179.44 (15)
C4—C5—C6—C1	−1.8 (3)	C7—C8—C13—N1	−4.2 (2)
C4—C5—C6—C7	177.37 (17)	C14—N1—C13—C12	−12.0 (3)
C1—C6—C7—O1	−154.66 (19)	C14—N1—C13—C8	167.63 (18)
C5—C6—C7—O1	26.2 (3)	C13—N1—C14—C15	177.31 (15)
C1—C6—C7—C8	25.1 (3)	N1—C14—C15—C16	−177.10 (18)
C5—C6—C7—C8	−154.06 (16)	N1—C14—C15—C20	1.7 (3)
O1—C7—C8—C9	57.0 (2)	C20—C15—C16—C17	−0.4 (3)
C6—C7—C8—C9	−122.72 (18)	C14—C15—C16—C17	178.42 (16)
O1—C7—C8—C13	−118.3 (2)	C15—C16—C17—C18	0.5 (3)
C6—C7—C8—C13	62.0 (2)	C16—C17—C18—C19	−0.3 (3)
C13—C8—C9—C10	−1.1 (3)	C16—C17—C18—Cl1	178.94 (14)
C7—C8—C9—C10	−176.41 (16)	C17—C18—C19—C20	0.0 (3)
C8—C9—C10—C11	1.4 (3)	Cl1—C18—C19—C20	−179.23 (15)
C8—C9—C10—Cl2	−178.96 (13)	C18—C19—C20—C15	0.1 (3)
C9—C10—C11—C12	−0.9 (3)	C16—C15—C20—C19	0.1 (3)
Cl2—C10—C11—C12	179.54 (14)	C14—C15—C20—C19	−178.70 (17)

### Hydrogen-bond geometry (Å, º)

**Table:**

*D*—H···*A*	*D*—H	H···*A*	*D*···*A*	*D*—H···*A*
C9—H9*A*···O1^i^	0.93	2.52	3.426 (2)	164

Symmetry code: (i) −*x*, −*y*, −*z*+2.
